# Efficacy and safety of systemic, high-dose glucocorticoid therapy for idiopathic sudden sensorineural hearing loss: Study protocol for a three-armed, randomized, triple-blind, multicenter trial (HODOKORT)

**DOI:** 10.1007/s00106-022-01184-8

**Published:** 2022-06-20

**Authors:** Stefan K. Plontke, Matthias Girndt, Christoph Meisner, Iris Böselt, Beatrice Ludwig-Kraus, Michael Richter, Torsten Rahne

**Affiliations:** 1 Department of Otorhinolaryngology, Head & Neck Surgery, Martin Luther University Halle-Wittenberg, Ernst-Grube-Str. 40, 06120 Halle (Saale), Germany; 2 Department of Medicine, Martin Luther University Halle-Wittenberg, Halle (Saale), Germany; 3 Robert Bosch Society for Medical Research, Robert Bosch Hospital, Stuttgart, Germany; 4 Coordination Centre for Clinical Trials, Martin Luther University Halle-Wittenberg, Halle (Saale), Germany; 5 Central Laboratory, University Hospital Halle, Halle (Saale), Germany

**Keywords:** Corticosteroids, Dexamethasone, Glucocorticoids, Humans, Idiopathic, Prednisolone, Randomized controlled trial, Steroids, Sudden hearing loss

## Abstract

**Background:**

Systemic glucocorticosteroids (“steroids”) are widely used worldwide as a standard of care for primary therapy of idiopathic sudden sensorineural hearing loss (ISSHL). The German ISSHL guideline recommends high-dose steroids without evidence from randomized controlled trials (RCTs) and refers solely to retrospective cohort studies. This RCT aims to assess the efficacy (improvement in hearing) and safety (especially systemic side effects) of high-dose steroids versus standard of care (standard dose systemic steroids) for the treatment of unilateral ISSHL, when given as a primary therapy.

**Methods:**

The study is designed as a multicenter (approximately 40 centers), randomized, triple-blind, three-armed, parallel group, clinical trial with 312 adult patients. The interventions consist of 5 days of 250 mg/day intravenous prednisolone (intervention 1) + oral placebo, or 5 days of 40 mg/day oral dexamethasone (intervention 2) + intravenous placebo. The control intervention consists of 60 mg oral prednisolone for 5 days followed by five tapering doses + intravenous placebo. The primary efficacy endpoint is the change in hearing threshold in the three most affected contiguous frequencies between 0.25 and 8 kHz 1 month after ISSHL. Secondary endpoints include further measures of hearing improvement including speech audiometry, tinnitus, quality of life, blood pressure, and altered glucose tolerance.

**Discussion:**

There is an unmet medical need for an effective medical therapy of ISSHL. Although sensorineural hearing impairment can be partially compensated by hearing aids or cochlear implants (CI), generic hearing is better than using hearing aids or CIs. Since adverse effects of a short course of high-dose systemic corticosteroids have not been documented with good evidence, the trial will improve knowledge on possible side effects in the different treatment arms with a focus on hyperglycemia and hypertension.

**Trial registration:**

EudraCT (European Union Drug Regulating Authorities Clinical Trials Database) Nr. 2015-002602-36; Sponsor code: KKSH-127.

## Administrative information

Note: the numbers in curly brackets in this protocol refer to SPIRIT checklist item numbers. The order of the items has been modified to group similar items [6, 12]. For administrative information see Table 1.

**Table 1:** Administrative information

Title {1}	Efficacy and safety of high-dose glucocorticoid treatment for idiopathic sudden sensorineural hearing loss—a three-armed, randomized, triple-blind, multicenter trial (HODOKORT)
Trial registration {2a and 2b}	EudraCT (European Union Drug Regulating Authorities Clinical Trials Database) Nr. 2015-002602-36; Sponsor code: KKSH-127; DRKS (German Clinical Trials Register): DRKS00010738
Protocol version {3}	Version: 03 Final, September 1, 2017
Funding {4}	This clinical trial is funded by the Federal Ministry of Education and Research (BMBF) in the grant program “Clinical trials of high relevance for patient care” within the German Federal Government’s “Health Research Framework Program” (funding code 01KG1427)
Author details {5a}	*Coordinating (chief) investigator:* Stefan K. Plontke *Deputy (chief) coordinating investigator:* Matthias Girndt *Responsible trial audiologist:* Torsten Rahne *Data management, monitoring, safety management:* Coordination Centre of Clinical Trials All: Martin Luther University Halle-Wittenberg, Halle (Saale), Germany *Biostatistics:* Christoph Meisner, Robert Bosch Society for Medical Research, Robert Bosch Hospital, Stuttgart, Germany
Name and contact information for the trial sponsor {5b}	Martin Luther University Halle-Wittenberg, Medical Faculty Magdeburger Straße 8, 06108 Halle, Germany
Role of sponsor {5c}	n/a: The funders have no role in the study design (apart from peer review during the funding institution’s grant application process); collection, management, analysis, and interpretation of data; writing of the report; and the decision to submit the report for publication, and they do not have ultimate authority over any of these activities

## Introduction

### Background and rationale {6a}

The World Health Organization states that over 5% of the world’s population—or 430 million people—require rehabilitation to address their ‘disabling’ hearing loss [43]. From over 1.5 billion people who experience some degree of hearing loss, which can significantly impact their lives, their families, society and countries, approximately one fourth have moderate-to-complete hearing loss in their better ear. Hearing loss is responsible for over 40 million years lived with disability (YLD) and was ranked as the third most common cause of YLDs in the Global Burden of Disease Study [8]. Most patients (> 80%) with hearing loss suffer from sensorineural hearing loss. Although sensorineural hearing impairment can be partially compensated by hearing aids (HA) or cochlear implants (CI), generic hearing is better than using hearing aids or CIs. With HAs and CIs, sound quality and communication ability are systematically reduced. In addition, tremendous costs for society are associated with communication disorders in general and with both HA and CI, specifically [44]. Besides age-related and drug- and noise-induced hearing loss, idiopathic sudden sensorineural hearing loss (in this protocol abbreviated as *ISSHL*, in other publications sometimes also abbreviated as *ISSNHL*) is one of the most frequent “causes” of sensorineural hearing loss. This clinical trial addresses hearing impairment due to ISSHL.

The incidence of sudden sensorineural hearing loss has been estimated to be 5–20 per 100,000 per year in industrialized countries [5]. However, according to studies in Germany, the incidence may be much higher: between 160 [27] and 400 per 100,000 per year [18]. The mean age of patients included in randomized controlled trials (RCTs) is between 45 and 55. Men and women are equally affected; ISSHL in childhood is rare.

Treatments of ISSHL with moderate-dose systemic glucocorticoids or other drugs have been assessed in RCTs, reviews, and Cochrane meta-analysis (e.g., [9, 19, 26, 40]), without demonstrating a clear efficacy of any of these therapies. However, systemic glucocorticoids are widely used as a standard of care for primary therapy of ISSHL worldwide [28]. For second-line (salvage, reserve) treatment conditions, but not for primary therapy of ISSHL, meta-analyses of RCTs suggest a possible advantage for locally (intratympanic) applied glucocorticoids The total number of patients in these studies, however, is low and the risk of bias is mostly high (e.g., [10, 14, 20, 24]).

The rationale for the treatment of ISSHL using high doses of systemic glucocorticoids is based on retrospective cohort studies. Alexiou et al. (2001) retrospectively analyzed the audiograms of 603 patients with ISSHL, with 301 patients (1986–1991) receiving no glucocorticoid and 302 patients (1992–1998) receiving high dose i.v. glucocorticoids (prednisolone) and showed a benefit for patients receiving high-dose prednisolone [1]. Egli Gallo et al. (2013) retrospectively evaluated the effectiveness of systemic high-dose dexamethasone therapy (administered orally) and demonstrated a significantly better improvement of hearing compared with a historical control using the clinic’s earlier treatment regimen (standard prednisone; [11]). Westerlaken et al. (2007), in a RCT, did not find a benefit of super-high-dose glucocorticoids compared with standard prednisolone [41]. Niedermeyer et al. (2003) showed in humans that inner ear cortisol levels were only increased after 250 mg of prednisolone (i.v.) but not after 125 mg i.v. [25]. The German ISSHL Guideline thus recommends high-dose glucocorticoid (250 mg prednisolone or equivalent glucocorticoid dose) for primary therapy of ISSHL [3], which, however, has not been proven so far in RCTs.

In general, possible side effects of systemic corticosteroid medication include metabolic complications, such as glucose intolerance and diabetes mellitus, hypertension, increased intraocular pressure and glaucoma, psychotropic effects, hypothalamic–pituitary–adrenal axis suppression, gastrointestinal bleeding, bone loss, avascular necrosis of the femoral or humeral head, and potential infections. A study investigating the risk of corticosteroid-induced hyperglycemia concluded that prevalence during systemic therapy is high and rises as the dose increases [33]. Although the rate of occurrence of side effects with systemic corticosteroid therapy for ISSHL appears to be low [15], systematic data-recording and publication of the proposed side effects are still insufficient, and adverse effects of a short course of high-dose systemic corticosteroids have not been documented with good evidence. It is only possible, therefore, to speculate whether these known side effects occur during systemic corticosteroid treatment of ISSHL and, if so, to what degree. Reduced glucose tolerance and enhanced blood glucose levels are typical complications of high-dose glucocorticoid treatment. A recent retrospective study of more than 2400 hospitalized patients who received systemic glucocorticoids found hyperglycemia in 36% [23]. A meta-analysis of former trials on this topic reported a similar rate of hyperglycemia upon glucocorticoid treatment (30%; [21]). Nevertheless, high-quality evidence on the effects of different doses and application schedules of glucocorticoids on glucose metabolism is lacking. Hyperglycemia is induced via several mechanisms, among them enhanced insulin resistance, increased gluconeogenesis, and reduced insulin secretion by the pancreatic beta cells [39]. Further, exacerbation of hypertension and de novo arterial hypertension are common consequences of glucocorticoid treatment. A population-based study recently documented that long-term glucocorticoid therapy enhances the risk of developing arterial hypertension in a dose-dependent manner [22]. Few data are available regarding immediate effects of high-dose glucocorticoids, and if studies addressed this topic, they often studied patients in whom glucocorticoids were applied for conditions such as glomerulonephritis that may already alter blood pressure as well. Therefore, this study addresses the question of whether different schedules and doses of glucocorticoids in the treatment of ISSHL may also have different side effects on glucose metabolism or blood pressure. This is an important topic since both complications have the potential to add morbidity and necessitate hospital admissions or prolonged hospital stays.

### Objectives {7}

To assess the efficacy (hearing improvement) and safety of high-dose systemic glucocorticoids (glucocorticosteroids) versus standard of care (standard-dose systemic glucocorticoids) for the treatment of unilateral idiopathic sudden sensorineural hearing loss (ISSHL), when given as a primary therapy.

### Trial design {8}

This is a three-armed, parallel-group, randomized, triple-blind, multicenter superiority trial.

## Methods: participants, interventions, and outcomes

### Study setting {9}

The study is carried out at approximately 40 study centers in Germany including academic hospitals, community hospitals, and private otorhinolaryngologic (outpatient) clinics throughout Germany. A list of study centers is available on the HODOKORT trial webpage (https://hodokort-studie.hno.org/studienzentren.html) of the German Study Centre for Otorhinolaryngology, Head and Neck Surgery (DSZ-HNO) or readers can refer to https://www.drks.de (ID DRKS00010738)*.*

### Eligibility criteria {10}

#### Inclusion criteria

- Informed consent
- Female and male adults (18–80 years)
- Unilateral sensorineural hearing loss
- Sudden onset of hearing loss (occurring within 24 h)
- Unknown etiology (no other ear or central nervous system disease)
- Change in hearing threshold due to ISSHL of 30 dB or higher for the three most affected contiguous frequencies in the affected ear in the frequency range of 0.25–8 kHz (as compared to a pre-event audiogram, the audiogram of the unaffected ear, or the DIN-ISO 7029)
- Absolute threshold of 50 dB HL or more as average of the three most affected contiguous frequencies in the affected ear in the frequency range of 0.25–8 kHz
- Enrolment and treatment within 7 days from onset
- Sufficient language comprehension to understand patient information and informed consent and for protocol adherence
- Contraception methods with Pearl Index less than 1%

#### Exclusion criteria

- Participation in another clinical trial in the last 30 days
- Recurrent ISSHL (ISSHL in the last 12 months at the affected side [ISSHL diagnosed and treated by an ENT specialist])
- Known systemic or other otologic cause of ISSHL (e.g., middle ear disease; known vestibular schwannoma [acoustic neuroma]; known fluctuating hearing loss; Meniere’s disease)
- Conductive hearing loss or conductive component (mixed hearing loss) with 4PTA_0.5–4_ _kHz_ > 10 dB and considering impedance audiometry
- Preexisting disease in the contralateral ear with known etiology, through which the cause of the hearing loss in the affected ear can be inferred
- Absolute threshold of less than 50 dB HL as average of the three most affected contiguous frequencies in the affected ear in the frequency range of 0.25–8 kHz
- In presence of any of the following diseases:
  - acute viral infection (herpes zoster, herpes simplex, varicella zoster, herpes keratitis)
  - HBsAg-positive chronic-active hepatitis
  - systemic mycosis and parasitosis (amoebiasis, helminthiasis)
  - poliomyelitis
  - lymphadenitis after BCG vaccination
  - acute and chronic bacterial infection
  - tuberculosis (current or in medical history)
  - severe osteoporosis
  - suicidal tendency (current or in medical history)
  - difficult-to-control hypertension
  - difficult-to-control diabetes
  - closed- or open-angle glaucoma, corneal erosion, or abrasion
  - severe ulcerative colitis with threatening perforation, abscesses, or purulent inflammation
  - diverticulitis
  - recent enteral anastomosis
  - stable angina, heart failure > NYHA II
  - treatment for gastric or duodenal ulcer within the last year
  - myasthenia gravis
  - hereditary problems of galactose intolerance, lactase deficiency, glucose-galactose malabsorption
- Uncontrolled hypertension (systolic > 180 mm Hg or diastolic > 100 mm Hg, measurement at screening)
- Psychiatric disorder or disease (current or in medical history, with inpatient or outpatient treatment, medical treatment, or psychotherapy): inclusion only after critical evaluation by investigator
- After surgery in the last 6 weeks: inclusion only after critical evaluation by investigator
- Current immunosuppressive therapy of rheumatic or chronic-inflammatory diseases
- Initial treatment of the ISSHL with glucocorticoids or hyperbaric oxygen (initial treatment with i.v. electrolyte solution or Ginkgo permitted)
- Currently ongoing glucocorticoid therapy (apart from local therapy, e.g., eye, skin)
- Currently ongoing treatment with following medication: coumarin derivates, cardioactive glycosides, atropine or other anticholinergics (local therapy permitted), praziquantel, chloroquine, hydroxychloroquine, mefloquine, immunosuppressive substances, ciclosporin
- Vaccination with live vaccines (planned within 8 weeks or within 2 weeks after vaccination)
- Hypersensitivity against prednisolone, prednisolone 21-hydrogensuccinat, dexamethasone
- Pregnancy and lactation
- Alcohol or drug abuse
- Other medical reasons that, after assessment by the investigator, are in conflict with inclusion

#### Inclusions of diabetics

Diabetics can be included in the study if they are well controlled and are hospitalized during the first five days. Additional blood glucose controls must be performed 4–6 hours after each administration of study medication on day 1–5. In case of critical blood glucose elevations (multiple > 10 mmol/L), an internal medicine consultation should be initiated. In addition, consultation with the trial chief investigator is recommended.

### Who will take informed consent? {26a}

Participation in the clinical trial is voluntary for all patients. All patients are informed verbally and in writing about the nature, significance, and scope of the study, the possible benefits and risks of the treatment, and the rights and responsibilities of study participants by an investigator. A patient information sheet in comprehensible/non-technical language will be handed out and there will have enough time for the individual to decide on whether to take part in the study as well as for clarification of any questions by a trial investigator.

### Additional consent provisions for collection and use of participant data and biological specimens {26b}

All patients are informed by the investigator about the transmission and use of data and biological specimens (blood samples) collected.

## Interventions

### Explanation for the choice of comparators {6b}

The control intervention is the most frequently used dose and considered international standard of care for systemic therapy of ISSHL (e.g., [26, 28, 32, 40]). According to the Declaration of Helsinki and to clinical experience, a placebo control is not acceptable and not feasible with respect to the patient’s expectance of treatment and ethical considerations. In Germany, a high-dose glucocorticoid therapy (250 mg prednisolone or equivalent glucocorticoid dose) is suggested in the German ISSHL guideline [3] without its effectiveness being proven in an RCT. Niedermeyer et al. (2003) showed in humans that inner ear cortisol levels were only increased after 250 mg of prednisolone (i.v.) but not after 125 mg (i.v.) [25]. The rationale for using a second, high-dose glucocorticoid treatment arm is based on the report of Egli Gallo et al. (2013), who retrospectively evaluated the effectiveness of systemic high-dose dexamethasone therapy (administered orally) and demonstrated a significantly better improvement of hearing compared with a historical control using the clinic’s earlier treatment regimen (standard prednisone [11]). Oral therapy would increase feasibility of the treatment for the patient compared with daily i.v. application and thus also demonstrates a patient-relevant outcome if shown to be effective.

### Intervention description {11a}

**Fig. 1:**
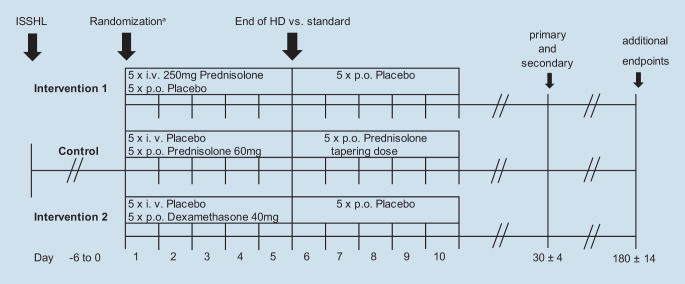
Intervention scheme. *HD* high dose, *ISSHL* idiopathic sudden sensorineural hearing loss, *i.v.* intravenous, *p.o.* per os. ^a^Randomization and first study medication intake/injection may be on same day

Our trial will compare both high-dose treatments with standard treatment in a confirmative manner (Fig. 1). There is no evidence for the necessity of dose reduction after short-term high-dose glucocorticoid therapy. Therefore, high-dose treatment is stopped without dose reduction over some days. All active trial drugs are commercially available. High-dose prednisolone (treatment intervention 1) is applied intravenously, while high-dose dexamethasone (treatment intervention 2) is given orally.

The participants will be randomized equally to one of the three treatment groups (one control, two interventions) according to the following treatment schemes:

- Intervention 1: 5 days of 250 mg/day prednisolone-21-hydrogen succinate, sodium salt intravenously (+ placebo p.o. 10 days)
- Intervention 2: 5 days of 40 mg/day dexamethasone orally (+ placebo i.v. 5 days/p.o. 5 days)
- Control: 60 mg prednisolone orally for 5 days followed by five tapering doses (+ placebo i.v. 5 days)

Patients will be followed up for 30 days after randomization for primary and secondary endpoints as well as safety parameters and for 6 months after randomization for additional secondary endpoints with relevance for patients.

### Criteria for discontinuing or modifying allocated interventions {11b}

The following stopping rules will apply:

- For the individual patient:
  In the case of adverse events, which are likely related to study medication and cannot be controlled appropriately (e.g., by anti-hypertensive medication in cases of high blood pressure), or in the case of any other adverse event that, according to the investigator’s assessment, will not enable continuation of treatment in the study.
  If a vestibular schwannoma is diagnosed during MRI examination.
  In the case of a pregnancy of the patient.
  After emergency unblinding.
  After withdrawal of informed consent.
  Lack of compliance by the participant.
- For the individual trial center:
  Lack of recruitment in the first 6 months after initiation.
  Dropout rate/lost to follow-up rate higher than 30%.
- For the whole trial:
  The sponsor may terminate the study prematurely if there are any relevant medical or ethical concerns or conducting the study is not feasible. Patients who are still under treatment at the time of termination must undergo a final examination, which will be documented accordingly. If an investigator has ethical concerns about continuation of the trial, he/she must inform the coordinating investigator immediately.

Premature termination of the clinical trial must be considered if:

- The benefit–risk ratio for the patient changes considerably.
- The application of the trial medication cannot be justified any longer.
- The sponsor considers a termination of the clinical trial due to safety issues (e.g., acting upon the advice of the DSMB).
- There is early proof of a superiority or inferiority of one of the treatment groups shown by an intermediate analysis or other research findings.
- The clinical trial does not prove feasible.

The sponsor will make the final decision about the termination of the clinical trial in consultation with the coordinating investigator, the DSMB, and the statistician.

### Strategies to improve adherence to interventions {11c}

Since the study drugs do not involve a completely new therapeutic concept, compliance of patients and sites to interventions is generally estimated as high. Comprehensive briefing at the pre-study and initiation visits and the continuous monitoring are measures to improve adherence. Additionally, sites are provided with, e.g., “pocket cards” and patients with a patient diary, which is checked by the investigators at each visit. A digital screening tool for checking the audiological inclusion criteria will be provided [31].

### Relevant concomitant care permitted or prohibited during the trial {11d}

Allowed concomitant medications are proton pump inhibitors (e.g., pantoprazole 2 × 20 mg/day).

Treatment not permitted during the trial is medication that has been used for the treatment of ISSHL in the past or is currently under investigation in clinical trials including: blood-flow-promoting drugs (rheologic drugs, vasodilators), antioxidants, medication- or non-medication-based fibrinogen reduction (e.g., batroxobin, H.E.L.P. apheresis, rheopheresis), drugs aimed at the reduction of endolymph volume (e.g., diuretics, mannitol), thrombocyte aggregation inhibitors, hyperbaric oxygen, antiviral drugs, NMDA-receptor antagonists, nootropics (e.g., piracetam [*Ginkgo biloba* is permitted]), calcium antagonists, magnesium, antiapoptotic drugs, and growth factors.

There is some evidence that (off-label) local, intratympanic glucocorticoid injection into the middle ear might be effective as a secondary (“rescue”) therapy after failure of primary systemic treatment. However, the total number of patients in these trials is small and the type of drug, dose, start of secondary treatment and application scheme, criteria of improvement, and baseline data vary between the studies and bias is high [10, 14, 20, 24, 38].

Between randomization and final visit (1 month), none of the aforementioned medication is permitted. For ethical reasons, alternative treatments (see above) are allowed after the 1‑month study period and will be recorded at the follow-up visit (at 6 months).

### Provisions for post-trial care {30}

For all patients participating in this trial, an insurance covering trial-related harms is contracted according to national law. Post-trial care will be at the investigator’s discretion and carried out according to the German treatment guidelines/clinical routine.

### Outcomes {12}

#### Primary outcome

There is no international consensus on the evaluation of outcome in the treatment of ISSHL (e.g., [29, 38]). The pure tone threshold is the internationally most standardized measure for hearing loss. The change in hearing threshold is used in most studies on sudden hearing loss or other inner ear disorders and was applied in high-quality RCTs on ISSHL [26, 32]. Pure tone threshold is correlated with hearing capability in quiet and noise [37]. Thus, it is relevant for everyday and working life. The WHO classifies hearing loss based on pure tone audiometry measurements and provides recommendations for the use of hearing aids or additional rehabilitation based on the pure tone audiometry-related grades [45]. This is also because, to date, pure tone audiometry is the measurement with the highest degree of international comparableness compared with speech audiometry in quiet and noise or with questionnaires. The data pool available for sample size calculation, based on national and international publications, is the largest for changes in pure tone threshold [9, 26, 28, 32].

Therefore, the *change in pure tone average of the three mostly affected contiguous frequencies* (between 0.25 and 8 kHz, i.e., 250 Hz, 500 Hz, 1 kHz, 1.5 kHz, 2 kHz, 3 kHz, 4 kHz, 6 kHz, 8 kHz) is measured as *the primary outcome after 30 days from the start of therapy*. Thresholds that were not measurable due to the limit of the audiometric equipment will be “dummy coded” with the highest level of the audiometric equipment. The 30-day time point has been chosen based on the results of the largest high-quality RCT to date [32], demonstrating no further change in pure tone threshold between 2 and 6 months and the available evidence of an effective intratympanic salvage therapy for sudden hearing loss [10, 14, 20, 24].

#### Secondary outcomes

Speech understanding (especially in noise) reflects a patient’s ability to communicate in everyday and working life better than pure tone threshold. However, the results of speech audiometry in different languages cannot easily be compared and are also influenced by other parameters such as speech competence. Speech audiometry tests are also difficult to interpret in patients whose native language is not that of the test lists.

Different categories of improvement based on change in pure tone or speech reception threshold have been suggested (e.g., [4, 13, 17, 36, 38] among others). A clinical practice guideline recognizes that fixed levels of improvement “… may have different benefits for different patients …” [38]. Therefore, speech audiometry and categories of improvement will be used as secondary outcome measures in this study. The recovery category is defined based on the pure tone average 30 days after start of therapy and referenced to the pre-event audiogram, the audiogram of the unaffected ear, or the ISO age- and sex-corrected standard audiogram: deterioration (> 10 dB increase, i.e., worsening), no change (≤ ±10 dB change), partial recovery (> 10 dB increase, i.e., improvement but not ≤ 10 dB difference to reference audiogram), and complete recovery (≤ 10 dB difference to reference audiogram). Most international trials and classifications of hearing improvement use 10 dB as a criterion for “partial improvement” (summary in [29], recommendation in [38]). The 10-dB criterion is based on the 5‑dB resolution of the method (ISO 8253-1), the test-retest reliability, and the clinical relevance of the hearing improvement.

As suggested by Stachler et al. [38] and others, for those patients with initial profound hearing loss (“non-serviceable hearing”) and partial recovery, it will be evaluated whether the hearing threshold resulted in “serviceable hearing,” meaning whether these patients would benefit from a conventional hearing aid. However, no international consensus exists with respect to hearing ability being “meaningful” or “serviceable.” There are different categories of hearing impairment. While the American Speech–Language–Hearing Association, according to Clark (1981), defines profound hearing loss as ≥ 91 dB, the WHO defines profound hearing loss with ≥ 80 dB pure tone average [7, 45]. There are no clear-cut criteria on whether a patient will benefit from using a hearing aid or not. The hearing level at which a hearing aid is useful covers a range of thresholds (see also WHO grades of hearing impairment [45]). Whether a hearing aid is used or not, and especially whether a cochlear implant is decided for, depends on a variety of objective and subjective factors, and on regional factors with respect to the world. However, since this is a patient-specific and socio-economically relevant aspect, the percentage of patients having received or being planned for rehabilitation with a hearing aid or a cochlear implant will be evaluated at a follow-up visit 6 months after ISSHL. In addition, subjective patient evaluation and quality of life will be assessed by means of the patients’ questionnaires that were selected in cooperation with three patient self-help groups (German Cochlear Implant Society [Deutsche Cochlear Implant Gesellschaft e.V., DCIG, Senden], German Alliance of Hearing Impaired [Deutscher Schwerhörigenbund e.V., DSB, Berlin], and German Society of Hearing Impaired [Deutsche Gesellschaft der Hörgeschädigten – Selbsthilfe und Fachverbände e.V., DG, Rendsburg]).

Arterial hypertension and impaired glucose tolerance are the two major adverse events following short-term high-dose glucocorticoid treatment. Alterations in glucose metabolism arise from enhanced hepatic gluconeogenesis and impaired glucose uptake by adipose tissues [35]. In healthy individuals, this rarely leads to relevant complications. However, in the presence of already impaired glucose tolerance, hyperglycemia may occur. The HOMA-IR is a useful index for detecting subclinical insulin resistance, which is more sensitive for detecting altered glucose metabolism than mere measurements of blood glucose [2].

Blood pressure effects of glucocorticoids are in part mediated by renal fluid retention and increased vascular responsiveness to catecholamines; however, additional unknown mechanisms seem to be involved [42]. Blood pressure elevations will be detected by 24‑h blood pressure measurements on day 5 of glucocorticoid treatment, where maximum effects are expected.

### Participant timeline {13}

For the time schedule of enrolment, interventions, assessments, and visits for participants, see Table 2.

**Table 2:** Time schedule of enrolment, interventions, assessments, and visits for participants

	BL^a^			Therapy			Th. end	FU 1^b^	FU 2^b^
Visit	1	2	3	4	5	6	7	8	9
Day	−6 to 1	1	2	3	4	5	10–13	30 ± 4	180 ± 14
*Demographic data*	X	-	-	-	-	-	-	-	-
*Informed consent*	X	-	-	-	-	-	-	-	-
*Inclusion and exclusion criteria, medical history*	X	-	-	-	-	-	-	-	-
*Physical examination*^1^	X	-	-	-	-	-	-	-	-
*Laboratory assessments*^2^									
Complete blood count	X	-	-	-	-	-	-	-	-
Clinical chemistry tests	X	-	-	-	-	-	-	-	-
Pregnancy test	X	-	-	-	-	-	-	-	-
Blood sample (fasting) for central laboratory^3^	X^3^	-	-	-	-	X^3^	-	-	-
Shipment of samples to central laboratory	-	-	-	-	-	X	-	-	-
POCT-glucose (before medication)	X^c^	X^c^	X	X	X	X	X	X	X
*Audiological assessments*^4^									
Pure tone audiometry	X^c^	X^c^	-	X^5^	-	X^5^	X	X	X
Impedance audiometry	X	-	-	-	-	-	-	X	X
Speech audiometry	X^c^	X^c^	-	X^5^	-	X^5^	X	X	X
*Otoneurological assessment*^6^	X		-	-	-	-	-	-	-
*Blood pressure, pulse*	X^c^	X^c^	X	X	X	X	X	X	X
*Long-term blood pressure*	-	-	-	-	-	X^7^	-	-	-
*Study medication*									
Documentation of concurrent medication	X	X	X	X	X	X	X	X	X
Application of study medication i.v.	-	X	X	X	X	X	-	-	-
Dispensing of p.o. medication and instruction	-	X	-	-	-	X	-	-	-
*Documentation of adverse events (AEs)*	-	X	X	X	X	X	X	X	-
*Documentation of quality of life (QOL)*	X^c^	-	-	-	-	-	-	X	X
*Documentation of tinnitus*	X^c^	-	-	-	-	-	-	X	X
*Indication for hearing aid/cochlear implant*	-	-	-	-	-	-	-	X	X
*MRI*	X								
*CRF-documentation/drug accountability*	X	X	X	X	X	X	X	X	X

*POCT* point-of-care testing, *Th. end* end of drug therapy

^a^ *BL* baseline

^b^ *FU* follow-up, FU 1: primary endpoint

^c^ Assessments at visits 1 & 2 can be made on the same day, e.g., the patient may receive the first study medication on the day of enrolment. Assessment of baseline blood glucose, pure tone and speech audiometry, blood pressure, pulse, quality of life and tinnitus should not take place more than 1 day before the first study medication

^1^ Includes: physical examination of all organ systems, weight, size, ENT examination, body temperature

^2^ Includes: complete blood count, clinical chemistry tests (ALAT, ASAT, GGT, ALP, creatinine, sodium, potassium, calcium, CRP), pregnancy test (hCG)

^3^ Central laboratory evaluations: measurement of HbA1c (baseline only), insulin, HOMA-IR (baseline and V6), fasting if possible, blood sample must be taken before medication; if V6 Saturday or Sunday or holiday: blood sample may be taken at last or next working day

^4^ Pure tone audiogram 250–8000 Hz, bone and air conduction; impedance audiometry, speech (monosyllables at 65 and 80 dB SPL with Freiburger speech test)

^5^ If visit on Saturday, Sunday or holiday these assessments may be omitted

^6^ Includes: spontaneous nystagmus, caloric or head impulse test, Romberg test; may be done at V1 or V2, at V2 also after medication possible

^7^ Day 5 ± 2 days

### Sample size {14}

This three-armed trial (randomization ratio: 1:1:1) is aimed at comparing standard-dose systemic therapy with two types of high doses of systemic therapy concerning the primary endpoint using two individual statistical tests assuming a normal distribution of the primary endpoint in the population. For a global significance level of 0.05, we adjust for two tests using a local level for each test of 0.025. The following assumptions are based on the available literature [32]. A sample size of 88 in each group will have 80% power to detect a difference between high dose and standard dose, when the true difference in means is −10.0 (e.g., the difference between a group 1 mean, μ1, of 30.7 and a group 2 mean, μ2, of 40.7) assuming that the common standard deviation is 21.3. The sample size calculation was performed for two-sample *t* tests with nQuery Version 7.0. Assuming a dropout rate of approximately 15%, a total of 312 patients need to be randomized (1:1:1) to maintain at least 264 evaluable patients with complete data (high-dose dexamethasone: 104; high-dose prednisolone: 104, standard prednisolone: 104).

Systemic glucocorticoids are the standard treatment in ISSHL. Since the study drugs thus do not involve a completely new therapeutic concept, patient compliance is generally estimated as high. We assume a rate of loss to follow-up of 15%. This estimate is based on two recent RCTs on ISSHL. Nosrati-Zarenoe and Hultrantz (2012) randomized 103 patients and were able to analyze 88 (85%) on day 8 of the assessment and 73 (71%) at the 3‑month time point [26]. In the study of Rauch et al. (2011), 221 of 255 patients (87%) completed the intervention and 2‑month follow-up (per-protocol analysis [32]).

The trial will be analyzed according to the intention-to-treat principle including all randomized patients. A per-protocol analysis will be performed as sensitivity analysis.

### Recruitment {15}

There has been no pilot study carried out using this design. The estimated recruitment rate of approximately 25% is based on three recent multicenter RCTs on ISSHL and on our own experience [17, 26, 30, 32]. Potentially participating centers were selected based on self-estimated recruitment: All centers were evaluated at pre-study visits. The German Professional Association of ENT Surgeons (BV-HNO e.V.) and the German Society of Otorhinolaryngology, Head & Neck Surgery e.V., Bonn, regularly remind German ENT specialists about the trial and the inclusion criteria through e‑mail newsletters and in the societies’ publications for their members. The study is also advertised through the website of the German Study Center for Otolaryngology, Head and Neck Surgery (DSZ-HNO) and the funders homepage. Study centers with no or insufficient recruitment will be closed.

## Assignment of interventions: allocation

### Sequence generation {16a}, concealment mechanism {16b}, and implementation {16c}

A computerized random algorithm using nQuery 7.0 was applied to generate the random allocation of the participants to one of the three study groups: group 1: i.v. prednisolone; group 2: i.v. dexamethasone; and group 3: oral prednisolone. To establish a balanced distribution of the therapy groups, a block-randomization with a fixed block size of 6 was used. The randomization was stratified for baseline PTA (< 81 dB vs. ≥ 81 dB, limit for profound hearing impairment according to WHO World Health Organisation 2008 Grades of Hearing Impairment). According to the randomization lists, an independent employee of the KKS Halle prepared packages including sets with study medication. Each package contained sets for three patients. The sets were numbered and looked equal irrespective of the mode of therapy included. The randomization details and the randomization lists were fixed in a separate document that is unavailable to anyone, involved in the enrolment, therapy application, data management, monitoring, and outcome assessments. The participating centers are centrally supplied with medication sets in such a way that each time a minimum of three sets are available. In the case of randomization, patients will be centrally assigned (information on the number of the study medication set) via an Internet-based automatic system by the Coordination Centre for Clinical Trials, University of Halle-Wittenberg, Halle (Saale).

## Assignment of interventions: blinding

### Who will be blinded {17a}

According to the allocation procedures (16a–c), the key study personnel and the patients were blinded. The authors of the final statistical analysis plan (e.g., principal investigators, statisticians) will be blinded concerning the therapy allocation of the patients but not to all other final data in order to establish a blinded review of the data before the start of the final analysis.

### Procedure for unblinding if needed {17b}

Unblinding is permissible in cases of emergency. Envelopes for emergency unblinding are available for the respective patient in the study medication package. Opening of an unblinding envelope must be documented.

## Data collection and management

### Plans for assessment and collection of outcomes {18a}

#### Hearing evaluation

##### Change in pure tone hearing threshold.

To evaluate the success of therapy, psychoacoustic hearing tests (audiometry) will be used to measure the change in the average pure tone hearing thresholds of the three adjacent audiometric frequencies (within a range of 0.25–8 kHz: 250 Hz, 500 Hz, 1 kHz, 1.5 kHz, 2 kHz, 3 kHz, 4 kHz, 6 kHz, 8 kHz) that are most affected by the ISSHL. The three most affected frequencies are identified by calculating the difference between the actual hearing threshold and the hearing threshold at the specific frequency from the reference audiogram. The reference audiogram is defined as (1) the pre-event audiogram, and, if not available (2) the audiogram of the unaffected ear, or, if also not available or applicable, (3) the ISO 7029 age- and sex-corrected standard audiogram. The three-frequency combination with the largest difference to the reference audiogram will be determined. The proportions of patients who completely recover, partially recover, do not at all recover their hearing, or with worsening of hearing will be determined.

##### Assessment of communication ability.

Speech intelligibility is measured specifically for each ear according to ISO 8253‑3 with two lists of the German Freiburger monosyllables test determining the percentage of monosyllables understood at 65 dB and 80 dB SPL, respectively. Furthermore, the proportion of patients who receive or are recommended to receive a conventional hearing aid or a cochlear implant after the treatment will be determined.

##### Assessment of quality of life.

Quality of life is assessed using the HHIE (Hearing Handicap Inventory for the Elderly) and SF 12 questionnaires, which must be completed by the patients themselves. This makes it clear to what extent the treatment in a therapy arm has led to a particular subjective improvement in the patient.

##### Tinnitus assessment.

The presence and severity of tinnitus are assessed with visual analog scales.

##### Evaluation of “rescue therapy.”

The proportion of patients who will receive any form of “rescue therapy” (outside the study protocol) is determined.

#### Assessments of systemic safety aspects

##### Blood pressure.

Long-term blood pressure measurement (24 h) is performed at the study centers on day 5 (±2 days). The pseudonymized measurement protocols (printout of the measured value lists) are transmitted to the coordinating center to enable evaluation under consideration of the technical quality. Therefore, the following criteria will be applied: at least nine complete measurements are required during daytime (06.00–22.00 h) and six during nighttime (22.00–06.00 h). If the standard deviation of the measurements in one of these time sections is > 25%, the measurements will be checked manually for validity. The circadian rhythm will be evaluated based on the difference between day- and nighttime average systolic pressure. If the nighttime average is ≥ 10% lower than the daytime average, the patient is categorized as a “dipper,” otherwise as a “non-dipper.”

##### Glucose tolerance.

To evaluate glucose tolerance, fasting capillary blood glucose and venous insulin are measured on the day before or the day of study inclusion as well as on day 5 of therapy. Homeostasis model assessment of insulin resistance (HOMA-IR) is computed with the following formula: HOMA-IR = fasting plasma glucose (FPG, mg/dL) × immunoreactive insulin (IRI, μIU/mL)/405 [34]. Serum samples from baseline and visit 6 are analyzed in the central laboratory in Halle (Saale), and blood glucose is measured locally.

For the time schedule of assessments and visits, see Table 2.

### Plans to promote participant retention and complete follow-up {18b}

Sites are trained on patient retention and the importance of completing follow-up. In addition to handing out patient diaries with scheduled visits, study sites are advised to remind patients of follow-up visits actively. In the case of premature termination of study treatment, the patient is asked for agreement with further surveys and the conducting of the follow-up visit. In the case of active withdrawal of consent (i.e., also for follow-up and documentation), the patient’s study participation is terminated, the conducting of further follow-up visits is no longer possible. The reasons for withdrawal of consent (if known) will be documented. The decision to withdraw consent from the study treatment must be without any disadvantage for the patient. Further treatment and follow-up outside the trial should be ensured. Potential loss of follow-up or non-compliance is considered in the statistical analysis plan.

### Data management {19}

Data are recorded directly at the trial sites by the study team in paper-based data collection (case report) forms (CRF) and forwarded to the data management of the Coordination Centre for Clinical Trials (KKS) Halle. In the KKS Halle, the data are continuously recorded and stored in the digital trial database. At an early stage, the accuracy of the data is checked by means of range, plausibility, and consistency checks, with implausible or missing data being corrected or added following requests/queries. The study management software secuTrial® (version 5.5.0.12; interActive Systems GmbH, Berlin, Germany) is used for data entry and query management—a validated, good clinical practice (GCP)-compliant database-supported complete solution for conducting clinical studies. All changes made to the data are stored in an audit trail. The study software has a study-specific adaptable user and role concept. The database is integrated into a general information technology (IT) infrastructure and security concept with a firewall and backup system. The data are backed up daily. The database will be closed once the input and the quality checks including blind review by the biometrician have been completed. The data are then forwarded for final evaluation to the responsible biometrician.

### Confidentiality {27}

Information about trial patients is kept confidential and managed under the applicable data protection laws and regulations. Access to the data is strictly limited to authorized persons.

The investigator and sponsor must ensure data protection of the patients. Patients must not be identified by names in any documents submitted to the sponsor. It will be ensured that all study materials and data are appropriately pseudonymized in accordance with data protection regulations prior to scientific use. Patients are informed that their study-related data will be stored in pseudonymized form. All data remain confidential and are subject to medical confidentiality. According to the national GCP regulation, these documents must and will be kept for at least 10 years.

### Plans for collection, laboratory evaluation, and storage of biological specimens for genetic or molecular analysis in this trial/future use {33}

n/a: The trial does not involve genetic or molecular analysis of biological specimens. No specimens will be stored for future use.

## Statistical methods

### Statistical methods for primary and secondary outcomes {20a}

The primary confirmatory analyses will be based on the intention-to-treat population (ITT), which will include all randomized patients. The analysis will be done according to the intention-to-treat principle; this means every patient remains in the treatment group as allocated by randomization. The per-protocol population (PP) will be formed from the ITT. Only patients with major protocol violations will be excluded. The details will be described in the final statistical analysis plan.

The PP will be the secondary analysis population for PP analysis to confirm the results of the ITT analysis.

The confirmatory analysis of the primary endpoint is planned after complete documentation and data cleaning including visit 9. The confirmatory analysis aims on showing the superiority of one or both of the high-dose treatments in comparison with the standard therapy. Therefore, two analyses of covariance (adjusted for baseline PTA) will be performed at a local significance level of α = 0.025 (two sided), which means for a global significance level of α = 0.05 (two sided) for the statistical evaluation of the primary endpoint.

The differences in the primary endpoint between the high-dose groups and the standard group will be estimated with 97.5% confidence intervals (being confirmatory and consistent to the test procedure) and with 95% confidence intervals (being comparable to results usually presented in the literature).

The sample size of the study was planned to achieve a high power for the comparisons of high dose vs. standard dose. But, if superiority of both high-dose treatments versus the standard treatment can be shown, both high-dose treatment arms will be compared at a significance level of α = 0.05 (two sided) and the difference will be estimated with 95% confidence interval as a secondary objective.

The data of the ITT and PP will be statistically described in detail. This includes:

- Listings of all documented data for every patient.
- Summary tables of all variables. Discrete variables will be described by absolute and relative frequencies. Continuous variables will be described by statistical characteristics (minimum, maximum, quartiles, median, mean, standard deviation).

The secondary endpoints will be analyzed as follows:

- Speech discrimination using Mann–Whitney tests as these values are usually not normally distributed.
- Comparison of patients with complete, partial, or no improvement will be compared with a Cochrane–Armitage test for trend.
- Data from QoL questionnaires using chi-squared tests and Mann–Whitney tests as adequate.

All statistical tests in connection to the secondary endpoints will be performed for descriptive purposes. The *p *values will be given without adjustment for multiple testing.

To evaluate the role of the baseline PTA as a prognostic or predictive factor for the primary and secondary endpoints, it is planned to analyze two subgroups according to baseline hearing loss (< 81 dB vs. ≥ 81 dB). The analysis will be the same as that for the primary intention-to-treat analysis including a test for interaction of baseline hearing loss and therapy.

Safety analyses will be performed in the safety population including all patients for whom one of the randomized treatments was started. Here, patients will be analyzed according to treatment received. Rates of adverse events and of serious adverse events will be calculated.

All confirmatory analyses were fully specified in the trial protocol. Other details of the statistical analysis will be fixed at the latest in the Statistical Analysis Plan, to be prepared during blind review before database lock.

### Interim analyses {21b}

An interim analysis for the primary and secondary outcomes is not planned.

### Methods for additional analyses (e.g., subgroup analyses) {20b}

To evaluate the role of the baseline PTA as a prognostic or predictive factor for the primary and secondary endpoints, it is planned to analyze two subgroups according to baseline hearing loss (< 81 dB vs. ≥ 81 dB). The analysis will be the same as that for the primary intention-to-treat analysis including a test for interaction of baseline hearing loss and therapy.

### Methods in analysis to handle protocol non-adherence and any statistical methods to handle missing data {20c}

The influence of missing audiological data on the results of the final analysis will be evaluated in a blind review of the structure of missing data and the reasons for missing data. Centers with more than 30% missing data at visit 9 will be suspended as recruiting center. The handling of missing data in the final analysis will be determined in the statistical analysis plan and will depend on the analysis of the structure of the missing data. We expect that the organizational structure of the trial will produce a missing-at-random structure. In this case, multiple imputation methods can be used to insert missing data for the analysis. In addition, sensitivity analysis will be carried out to evaluate the influence of missing data.

### Plans to give access to the full protocol, participant level-data, and statistical code {31c}

According to the recommendations on data sharing by the International Committee of Medical Journal Editors (ICMJE), data resulting from the study will be made available to the scientific community as follows:

After publication of the major results and upon reasonable request from researchers performing an individual patient data meta-analysis, individual patient data that underlie published results will be shared after de-identification.

Summary statistics that go beyond the scope of published material will be made available to researchers for meta-analysis upon reasonable request and if the necessary data analysis is not unduly time-consuming. Together with publication of the main results, the study protocol in full will be made publicly available as well as the statistical analysis plan.

## Oversight and monitoring

### Composition of the coordinating center and trial steering committee {5d}

Study coordination, data management, monitoring, and safety management are done by the Coordination Center for Clinical Trials at University Medicine Halle (KKS Halle), which has comprehensive experience with managing drug trials. Tasks assigned to KKS Halle will be performed according to written standard operating procedures (SOPs).

### Composition of the data monitoring committee, its role, and reporting structure {21a}

To provide expert advice and independent trial monitoring, a Data and Safety Monitoring Board (DSMB or data monitoring committee, DMC) is established. The DSMB follows the progress of the clinical trial, evaluates the safety parameters, and proposes to the sponsor whether to continue, modify, or stop a trial. Prior to inclusion of the first patient, a DSMB charter will be established by specifying roles and responsibilities as well as meeting frequency and reviewed data. The DSMB sets up conference calls twice a year to evaluate current safety data. Therefore, the sponsor will provide up-to-date safety data including a summary of adverse events and line listing of serious adverse events. The DSMB will inform the sponsor about their recommendation for trial continuation/discontinuation. In the case of a high number of severe unexpected events in between DSMB meetings or a case of a suspected unexpected serious adverse reaction (SUSAR) or other medically important conditions, the DSMB will be informed by the sponsor immediately and may give advice for further procedures if required.

### Adverse event reporting and harms {22}

Clinical safety management will be performed according to ICH E2A guideline. Adverse events are to be reported in the CRF. Reporting of serious adverse events will follow guidance ENTR/CT 3 (Detailed guidance on the collection, verification and presentation of adverse reaction reports arising from clinical trials on medicinal products for human use). All adverse events occurring within 26 days of the initial administration of study medication (visit 8 = days 26–34) are to be documented as adverse events. If an adverse event meets one of the seriousness criteria, additionally expedited reporting to the sponsor applies. Sites are provided with a detailed safety manual. The sponsor reports safety issues further according to protocol and national laws. Safety analyses will be performed in the safety population including all patients for whom one of the randomized treatments was started. Here, patients will be analyzed according to treatment received. Rates of adverse events and of serious adverse events will be calculated.

### Frequency and plans for auditing trial conduct {23}

As a measure of quality control, risk-adapted on-site data monitoring is conducted during the enrolment period by independent clinical monitors from the KKS Halle and the ZKS Freiburg (Clinical Trials Unit of the Medical Center—University of Freiburg) to ensure patient safety, adherence to protocol and GCP, and consistency of the data. This is done according to GCP and SOPs. The specific extent of the monitoring and source data verification is specified in the monitoring manual. Every trial site will undergo a close-out visit by the monitors after the last participant at that site has finished the follow-up visit. In compliance with GCP guidelines, further audits may be performed as a quality measure by the sponsor or an independent external party, as well as inspections by regulatory authorities. To date, one of the trial sites and the KKS Halle have been inspected by responsible governmental authorities.

### Plans for communicating important protocol amendments to relevant parties (e.g., trial participants, ethical committees) {25}

Any protocol modifications are communicated to relevant parties including investigators and regulators. Substantial amendments that require approval according to the national GCP regulation (GCP‑V §10) will be submitted to the responsible ethics committee and the relevant federal authority for evaluation and are only implemented after their approval.

## Dissemination plans {31a}

The trial was registered with full description at the German Clinical Trials Register (*Deutsches Register Klinischer Studien*, DRKS). The results will be published independent of the size or direction of the effects, according to CONSORT guidelines, in peer-reviewed national and international journals with a special emphasis on media that is relevant for professionals in otorhinolaryngology. Beyond regular journal publication, results will be presented and discussed at national and international congresses addressing ENT specialists and specialists for internal medicine. Furthermore, the resulting evidence may be implemented into the German (and other) ISSHL guideline(s). Additionally, clinical trial summary results will be published in the European Union Clinical Trials Database (EudraCT). Relevant raw data will be made available in a data repository. Study results will also be published in the respective publications of patient self-help organizations. To enhance patient interest and support, we will provide information in lay language via specific publications on our homepages, printed flyers, and regular oral presentations at local and national meetings of patient organizations in Germany. Moreover, we will be in direct contact and interaction with healthcare providers and regulatory governmental authorities to support their decision-making processes concerning the use of high-dose glucocorticoids in clinical practice and cost reimbursement.

## Discussion

There is an unmet medical need for an effective medical therapy of ISSHL. In this RCT we investigate the efficacy (improvement in hearing) and safety (especially systemic side effects) of high-dose steroids versus standard of care (standard-dose systemic steroids) for the treatment of unilateral ISSHL, when given as a primary therapy. However, there are several challenges associated with RCTs on ISSHL. Since systemic moderate doses of glucocorticoids for the treatment of ISSHL are considered a “standard of care” in most countries around the world, a placebo arm appears to be neither ethically justified nor practically possible due to patient expectations for treatment (usually with steroids). In addition, there is no international consensus on outcome criteria in ISSHL trials.

Determining a clinically relevant or important change in hearing is challenging. In this clinical trial, we have taken a change in hearing threshold of 10 dB HL to represent the minimally important difference (MID). However, we acknowledge that this may not be universally accepted. The decision to choose 10 dB as an MID was based on the test–retest reliability of pure tone audiometric measurements, on established minimal criteria for improvement in individual patients [16, 38], and on a large RCT on this topic with low bias [32].

The clinical relevance of this MID, however, depends on the degree of initial hearing loss (i.e., moderate, severe, profound hearing loss) and whether the patients had serviceable hearing before and/or after therapy [38]. For example, a 10-dB change might not be useful in severe or profound hearing loss if the patient (or the ear) would remain at a cochlear implant candidate level after therapy. The U.S. clinical practise guideline therefore correctly recommends that future studies should report the number of patients reaching serviceable hearing: “… For ears that were rendered nonserviceable by the episode of SSNHL, return to serviceable hearing should be considered a significant improvement, and whether or not this level of recovery occurs should be recorded. Recovery to a serviceable level typically indicates that after recovery, the ear would be a candidate for traditional hearing amplification. Recovery to less than serviceable levels indicates an ear that would in most circumstances not benefit from traditional amplification. For ears with SSNHL to hearing levels that are still in the serviceable range, a 10 dB HL improvement in pure-tone thresholds (the smallest recordable improvement outside of the range of error for most audiograms) or an improvement in WRS of ≥ 10% (approximate lower limit for a statistically significant change based on binomial tables for WRS of > 50% at baseline) should be considered partial recovery and recorded …” (cited from [38]).

Speech audiometry in quiet and noise is preferable over pure tone threshold measurements (or at least as a complementary measure) since they represent communication capabilities better. In German-speaking countries, speech recognition in quiet is typically measured at fixed sound pressure levels including 65 dB SPL (monosyllable word recognition at 65 dB SPL: WRS_65_). At approximately this level, every-day conversation takes place. Since this method is not adaptive to the speech reception threshold (SRT), e.g., such as a “40-dB above speech reception threshold” measure (40 dB SL as recommended by Gurgel et al., 2012, [16]), it allows for a direct assessment of communication abilities and is therefore included in the trial protocol as a secondary outcome measure. The maximum word recognition score (WRS_max_) would also be of interest for the assessment of options and audiological indications for hearing rehabilitation (e.g., conventional hearing aids or cochlear implants). However, the trial population involves patients with an acute “inner ear event.” Therefore, speech audiometry at high levels to determine the WRS_max_ was considered not appropriate and thus not included as an outcome measure. Speech perception in noise can, for instance, be measured in matrix tests that are already available in many languages. These tests, however, are rather time consuming and therefore less practical in the setting of the treatment of an acute event. In addition, they are not available in all study centers. Speech audiometry in noise was therefore not included as an outcome measure in this RCT.

This is the English version for journal publication according to the SPIRIT guidelines based on the full final study protocol (Version: 03 Final, September 1, 2017) available in German language. Some information (e.g., epidemiological data and background information) was updated in the introduction section of this manuscript.

Patient recruitment was stopped on March 27, 2020, due to the COVID-19 pandemic shortly before the planned number of patients was reached. The main reasons for stopping patient recruitment were (1) the (at that time) unknown effect of high-dose systemic glucocorticoid therapy on the course of an infection with the severe acute respiratory syndrome coronavirus type 2 (SARS-CoV-2), (2) the reduction of patient contacts to absolutely necessary medical and surgical treatments during the pandemic, (3) the expected difficulties in appropriate monitoring and quality control due to the restrictions in mobility, and (4) the very small number of remaining patients to complete the trial as planned (325 of 329 patients were already enrolled, with 17 patients with a vestibular schwannoma excluded). The coordinating/chief investigator and the sponsor made the final decision about the termination of patient recruitment after consultation with the DSMB, the deputy coordinating investigator, and the trial statistician. Despite the premature stop, the recruitment of the study can be considered successfully completed with 99% of the required patients.

## Trial status

Protocol version: 03 Final, September 1, 2017 (starting version 02 Final, May 27, 2016)

Start of BMBF funding: 01.04.2015

Approval of competent authority (BfArM): 17.06.2016

Approval of leading ethics committee: 21.06.2016

First patient in: 28.11.2016

Last patient in: 24.03.2020

Last patient out: 30.09.2020

Planned end of trial: end of trial is defined as the time point the data bank is locked.

Current status: “blind review” of the data by the responsible trial statistician before unblinding the treatment groups, in parallel with finalizing the statistical analysis plan (SAP).

## Declarations

### Acknowledgements

This clinical trial was funded by the Federal Ministry of Education and Research (BMBF) in the grant program “Clinical trials of high relevance for patient care” within the German Federal Government’s “Health Research Framework Program” (funding code 01KG1427; to S. K. Plontke).

The authors acknowledge the support of the following colleagues for making this trial possible (in alphabetical order): Gabriele Dreier (formerly at: German Study Center for Otolaryngology, Head and Neck Surgery [DSZ-HNO] Bonn); Roland Laszig (University Hospital Freiburg, former President German Society of Oto-Rhino-Laryngology, Head and Neck Surgery); Ilka Oerlecke, (formerly at: Coordination Centre for Clinical Trials, Martin Luther University Halle-Wittenberg); Rudolf Probst (University Hospital Zurich, Switzerland); Jörg Steighardt (Director: Coordination Centre for Clinical Trials, Martin Luther University Halle-Wittenberg); Jochen A. Werner (former director of the German Study Center for Otolaryngology, Head and Neck Surgery [DSZ-HNO] Bonn). Jan Löhler, Scientific Institute for Applied Oto-Rhino-Laryngology (WIAHNO) of the German Professional Association of ENT-Surgeons (BV-HNO), Bad Bramstedt, supported the proposal with respect to strategical and recruitment planning.

We acknowledge the team of pharmaceutical development of mibe GmbH Arzneimittel, Brehna (Dermapharm AG, Grünwald) for providing the study medication including the development and production of placebo medication on the basis of a contract between the sponsor and the company. For advice with respect to specific secondary outcome parameters, we thank the following colleagues (in alphabetical order): Ingo Baumann (QOL), Department of Otorhinolaryngology, Head & Neck Surgery, University of Heidelberg; Berthold Langguth (tinnitus), Department of Psychiatry and Psychotherapy and Tinnitus Center, University of Regensburg; Veronica Vielsmeier (tinnitus), Department of Otorhinolaryngology, Head & Neck Surgery and Tinnitus Center, University of Regensburg; Andreas Weber (QOL), formerly at: Institute for Health and Nursing Sciences, Medical Faculty, Martin Luther University Halle-Wittenberg. Roland Zeh, German Cochlear Implant Society [Deutsche Cochlear Implant Gesellschaft e.V., DCIG, Senden] and representatives from patient organizations (German Alliance of Hearing Impaired [Deutscher Schwerhörigenbund e.V., DSB, Berlin], German Society of Hearing Impaired [Deutsche Gesellschaft der Hörgeschädigten – Selbsthilfe und Fachverbände e.V., DG, Rendsburg]) provided advice in the choice of patient relevant outcome measures (PROM).

The Clinical Trials Unit of the Medical Center—University of Freiburg (ZKS Freiburg) supported the study with monitoring study centers in Southern Germany through a cooperation with the KKS Halle.

We also thank the members of the Independent Data and Safety Monitoring Board (DSMB, also: Data Monitoring Committee, DMC) Ralph Mösges, ClinCompetence Cologne GmbH, formerly at: Institute for Medical Informatics, University of Cologne; Claudia Schmoor, Biometry and Data Management, ZKS Freiburg; Martin Fassnacht, Dept. of Medicine I, University Hospital Würzburg.

(All in Germany if not otherwise mentioned).

### Author contributions {31b}

SKP conceived of the study, led the proposal and protocol development. TR, MG, CM, BLK, MR, and IB contributed to study design and development of the grant proposal and protocol. CM is the main trial biometrician. BLK provided expertise in laboratory analysis of safety parameters. All authors read and approved the final manuscript. SKP is the trial grant recipient and the chief investigator.

### Funding {4}

This clinical trial was funded by the Federal Ministry of Education and Research (BMBF) in the grant program “Clinical trials of high relevance for patient care” within the German Federal Government’s “Health Research Framework Program” (funding code 01KG1427 to S. K. Plontke). The funding body has no role in the design of the study (apart from the peer review process during application for funding), in collection, analysis, and interpretation of data and in writing the manuscript.

### Availability of data and materials {29}

The sponsor has access to the complete set of raw data of the trial. Each trial site will have access to their CRF data. The final trial dataset can be accessed upon relevant ethical approval by contacting the corresponding author on reasonable request. Relevant raw data will be made available in an open data repository.

### Ethics approval and consent to participate {24}

This clinical trial was approved by the German Competent Authority BfArM and by the leading independent Ethics Committee of the Medical Faculty, Martin Luther University Halle-Wittenberg, Germany (approval number 2016–33), in consultation with the local ethics committees responsible for the individual sites. Written, informed consent to participate is obtained from all participants.

### Consent for publication {32}

Consent forms as well as the study and medical information given to the participants are in the German language and can be accessed by contacting the corresponding author on reasonable request.

### Competing interests {28}

The authors declare the following competing interests:

**SKP:** AudioCure Pharma GmbH, Berlin, Germany (consultant, scientific advisory board); MED-EL, Austria and Germany (grant support to university, travel support and honoraria for lectures); Merck Serono, Darmstadt, Germany (honoraria for session moderation); Infectopharm, Heppenheim, Germany (honoraria for lectures); Cochlear Corp., Australia (grant support to university); Oticon, Denmark (grant support to university); German Professional Association of ENT-physicians, BV-HNO e.V. (honoraria for lectures).

**MG:** Amgen GmbH (scientific advisory board, lecture honoraria), Astellas GmbH (session moderator honoraria), Bayer Vital AG (lecture honoraria), Daiichi Sankyo GmbH (clinical study grant), Novartis GmbH (lecture honoraria), Sanofi GmbH (session moderator and lecture honoraria), Vifor Pharma GmbH (lecture honoraria).

**CM:** AudioCure Pharma GmbH, Berlin, Germany (consultant).

**IB, MR, BLK:** The authors declare that they have no competing interests.

**TR:** AudioCure Pharma GmbH, Berlin, Germany (consultant); MED-EL, Austria and Germany (grant support to university and travel support); Cochlear Corp., Australia (grant support to university and travel support); Oticon, Denmark (grant support to university and travel support).

### Authors’ information (optional)

**SKP **is member of the executive board and was president (2020/2021) of the German Society of Otorhinolaryngology, Head and Neck Surgery and is member of the steering committee of the German Study Center for Otorhinolaryngology, Head and Neck Surgery.

**MG **is member of the extended executive board of the German Society for Nephrology.

**TR** is vice president of the ADANO (workgroup of German-speaking audiologists, otologists and neurotologists) of the German Society of Otorhinolaryngology, Head and Neck Surgery and is member of the German Society of Audiology (DGA).

## Abbreviations

AE: Adverse event
ALAT: Alanine aminotransferase
ALP: Alkaline phosphatase
ASAT: Aspartate aminotransferase
BfArM: *Bundesinstitut für Arzneimittel und Medizinprodukte*, Federal Institute for Drugs and Medical Devices
CRF: Case report form
CRP: C‑reactive protein
DSMB: Data and Safety Monitoring Board (also: data monitoring committee, DMC)
ENT: Ear, nose, and throat
FPG: Fasting plasma glucose
GCP: Good clinical practice
GGT: Gamma-glutamyltransferase
HbA1c: Glycated hemoglobin
hCG: Human chorionic gonadotropin
HOMA-IR: Homeostatic model assessment—insulin resistance
IRI: Immunoreactive insulin
ISO: International Organization for Standardization
i.v.: Intravenous
KKS Halle: *Koordinierungszentrum für klinische Studien*, Coordination Center for Clinical Trials University Medicine Halle
MID: Minimally important difference
MRI: Magnetic resonance imaging
p.o.: Per os
POCT: Point-of-care testing
PTA: Pure tone average
QOL: Quality of life
SAE: Serious adverse event
SAP: Statistical analysis plan
SL: Sensation level
SOP: Standard operating procedures
SPL: Sound pressure level
SUSAR: Suspected unexpected serious adverse reaction
WRS: Word recognition score
ZKS: *Zentrum Klinische Studien*, Clinical Trials Unit of the Medical Center—University of Freiburg
